# Supplementary material for: Increased neuronal signatures of targeted memory reactivation during slow-wave up states
Source: Sci Rep. 2019 Feb 25;9:2715. doi: 10.1038/s41598-019-39178-2 (PMC6389952; doi:10.1038/s41598-019-39178-2)
Supplement: Supplementary file 1 — Supplementary Information [file 41598_2019_39178_MOESM1_ESM.pdf]

Supplementary Information

for

Increased neuronal signatures of targeted memory reactivation during slow-wave up states

Maurice Göldi[1,2], Eva van Poppel[1], Björn Rasch[1*], Thomas Schreiner[3*]

[1] University of Fribourg, Department of Psychology, Fribourg, Switzerland
[2] Neuroscience Center Zurich (ZNZ), Zurich, Switzerland
[3] Centre for Human Brain Health, School of Psychology, University of Birmingham, Birmingham, UK

Maurice Göldi, University of Fribourg, Department of Psychology, Division of Cognitive Biopsychology and Methods, Rue P.-A.-Faucigny 2, CH-1701 Fribourg, Switzerland

Eva van Poppel, , University of Fribourg, Department of Psychology, Division of Cognitive Biopsychology and Methods, Rue P.-A.-Faucigny 2, CH-1701 Fribourg, Switzerland

*Corresponding authors:
University of Birmingham, School of Psychology, Edgbaston, B152TT Birmingham, United Kingdom
Email: T.Schreiner@bham.ac.uk
Phone: +44 121 414 8690

Björn Rasch, University of Fribourg, Department of Psychology, Division of Cognitive Biopsychology and Methods, Rue P.-A.-Faucigny 2, CH-1701 Fribourg, Switzerland;
Email: Bjoern.Rasch@unifr.ch
Phone: +41 26 300 7637

Supplementary Table 1: Overview of memory performance

	Up-state	Down-state	Uncued
Learning	19.87 ± 0.90	20.00 ± 0.95	20.56 ± 0.94
Retrieval	19.75 ± 1.05	19.37 ± 1.21	18.87 ± 1.30
Change	- 0.12 ± 0.52	- 0.62 ± 0.80	- 1.68 ± 0.61
% Change	99.30 ± 2.89	90.92 ± 3.14	96.83 ± 4.27

Data are means ± SEM; Numbers indicate absolute or relative values of correctly recalled words that where either presented during SOs up- and down-states or remained uncued. For cued recall testing, the number of correctly recalled words during the learning phase before and the retrieval phase after the retention interval are indicated. Change (% Change) refers to the absolute (relative) difference in performance between the learning and retrieval phases.

Supplementary Table 2: Word list used in the memory task

Dutch	German	English
aap	Affe	monkey
baan	Beruf	job
beek	Bach	brook
been	Bein	leg
bel	Klingel	bell
beurs	Börse	stock market
bij	Biene	bee
blik	Blech	sheet metal
bloem	Blume	flower
bloes	Bluse	blouse
boek	Buch	book
boer	Bauer	farmer
bol	Kugel	sphere
boom	Baum	tree
bos	Wald	forest
bout	Bolzen	bolt
brug	Brücke	bridge
buik	Bauch	belly
buks	Büchse	rifle
dak	Dach	roof
deel	Teil	part
deur	Tür	door
dief	Dieb	thief
dijk	Teich	pond
doek	Tuch	cloth
dorp	Dorf	village
duim	Daumen	thumb
eed	Eid	oath
fles	Flasche	bottle
fout	Fehler	fault
geur	Geruch	odor
gif	Gift	poison
hak	Absatz	heel
hei	Heide	heath
hiel	Ferse	heel
hout	Holz	wood
hulp	Hilfe	help
hut	Hütte	hut
inkt	Tinte	ink
jas	Jacke	jacket
kast	Schrank	closet
kerk	Kirche	church
kok	Koch	cook
kras	Kratzer	scratch
kruk	Krücke	crutch
kus	Kuss	kiss
kust	Küste	coast
kwal	Qualle	jellyfish
lens	Linse	lens
lijf	Leib	body
lijm	Kleber	glue
lip	Lippe	lip
loof	Laub	foliage
melk	Milch	milk
mes	Messer	knife
mond	Mund	mouth
mug	Mücke	mosquito
muts	Mütze	cap
muur	Mauer	wall
neef	Neffe	nephew
neus	Nase	nose
nier	Niere	kidney
oog	Auge	eye
pad	Pfad	path
piek	Gipfel	peak
pijn	Schmerzen	pain
pijp	Pfeife	pipe
pols	Puls	pulse
pont	Fähre	ferry
prik	Spritze	syringe
rek	Regal	rack
rib	Rippe	rib
rijst	Reis	rice
rit	Fahrt	drive
roer	Ruder	rudder
rug	Rücken	back
rups	Raupe	caterpillar
sap	Saft	juice
schok	Schlag	blow
schol	Scholle	plaice
sla	Salat	salad
slot	Schloss	lock
sluis	Schleuse	sluice
snor	Schnurrbart	moustache
soep	Suppe	soup
spaak	Speiche	spoke
steen	Stein	stone
ster	Stern	star
stof	Staub	dust
stoot	Stoss	push
strijd	Kampf	battle
stuur	Lenkrad	steering wheel
taart	Kuchen	pie
tand	Zahn	tooth
tas	Tasche	bag
teek	Zecke	tick
tent	Zelt	tent
tijd	Zeit	time
tong	Zunge	tongue
trap	Treppe	stairs
veer	Feder	feather
vis	Fisch	fish
vlees	Fleisch	meat
voet	Fuss	foot
vork	Gabel	fork
vuil	Schmutz	dirt
vuur	Feuer	fire
waard	Wirt	innkeeper
walm	Qualm	smoke
will	Wille	will
winst	Gewinn	profit
wol	Wolle	wool
wond	Wunde	wound
worst	Wurst	sausage
zalm	Lachs	salmon
zeep	Seife	soap
zit	Sitz	seat
zon	Sonne	sun
zool	Sohle	sole
zout	Salz	salt

Wordlist used for memory task. Dutch-German word pairs used during the memory task.

Supplementary Figure 1: Slow Wave Detection Algorithm State Diagram

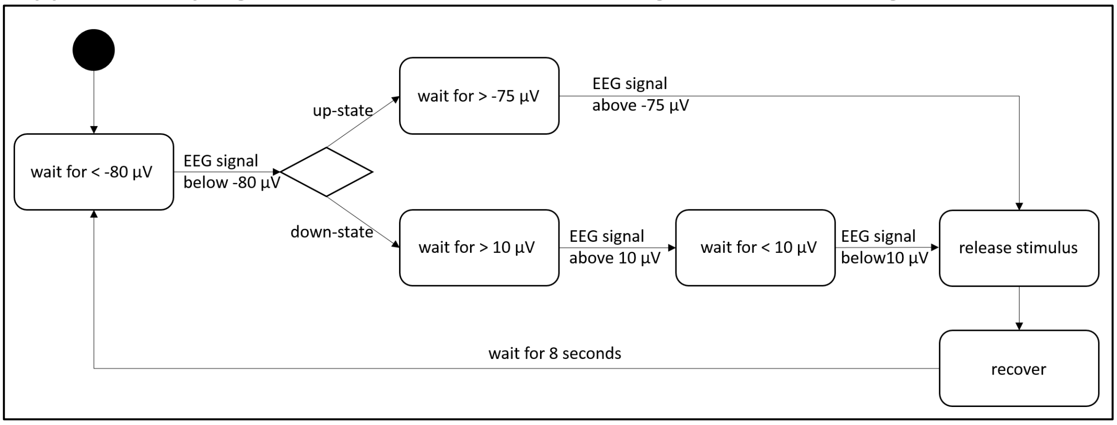

Online Detection Finite-State Machine Diagram Implementation of the slow-wave detection algorithm as a finite state machine. The algorithm starts at the black dot and traverses through the states while it is running.

Supplementary Figure 2: ERPs for Remembered and Non-Remembered cues

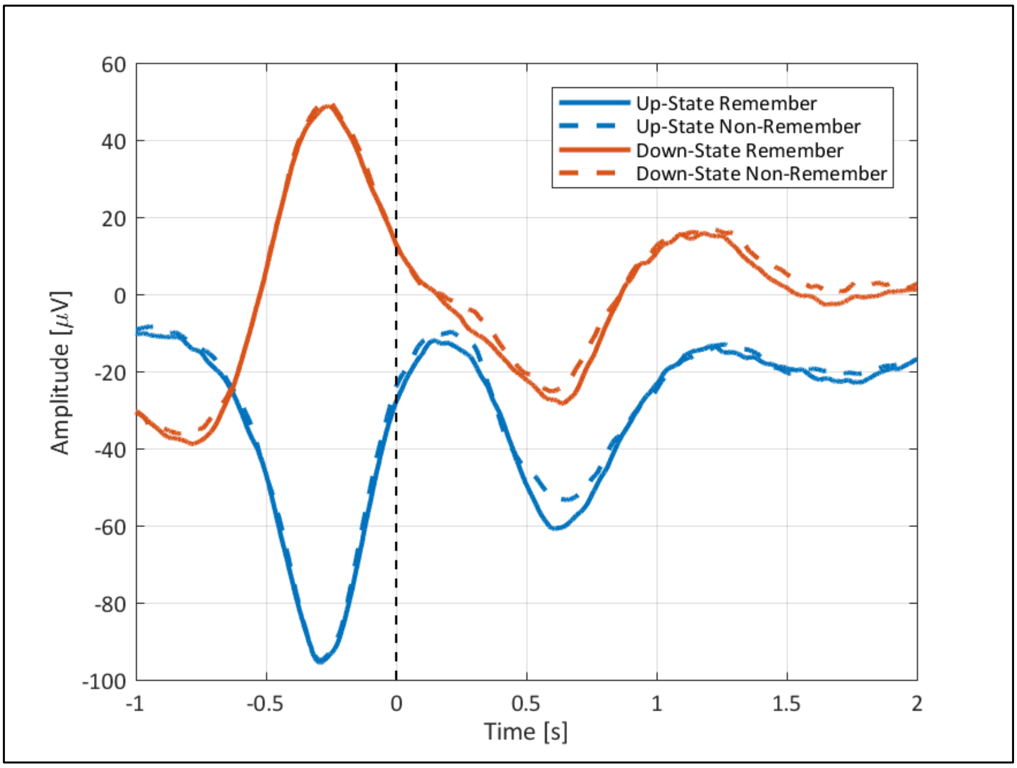

Comparison of ERPs for remembered and non-remembered words. ERPs for up- (blue) and down-state (red) remembered (solid line) and non-remembered (dashed line) words are shown. There is no significant difference between remembered and non-remembered word cues.

Supplementary Figure 3: Phase Distribution across the Scalp

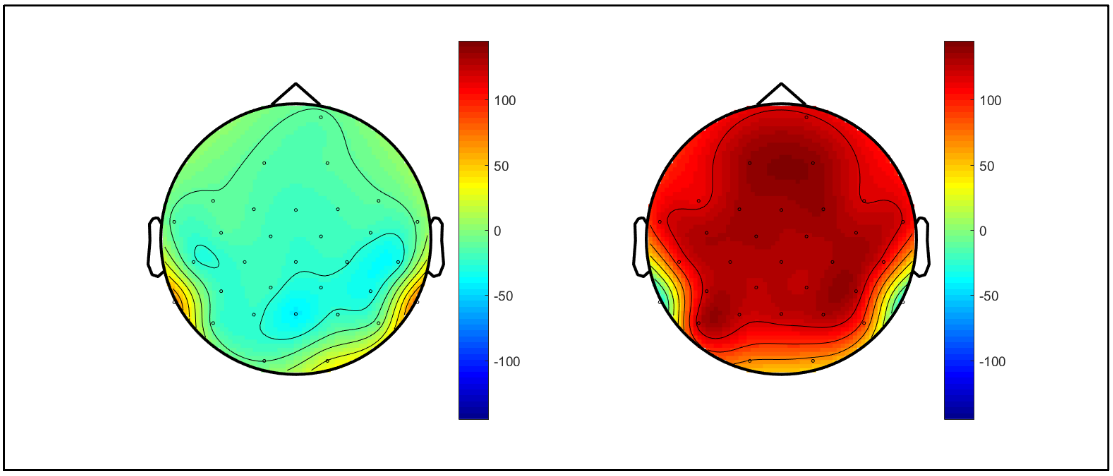

Topographical distribution of phase. Signal phase at stimulus release for up-state cues (left) and down-state cues (right). While the algorithm detects slow-waves at the Fz electrode only, the phase distribution across the scalp is uniform at the time of cue onset. Up-state phase is around -20° and down-state phase around 120°.

Supplementary Figure 4: Phase Accuracy for each Subject

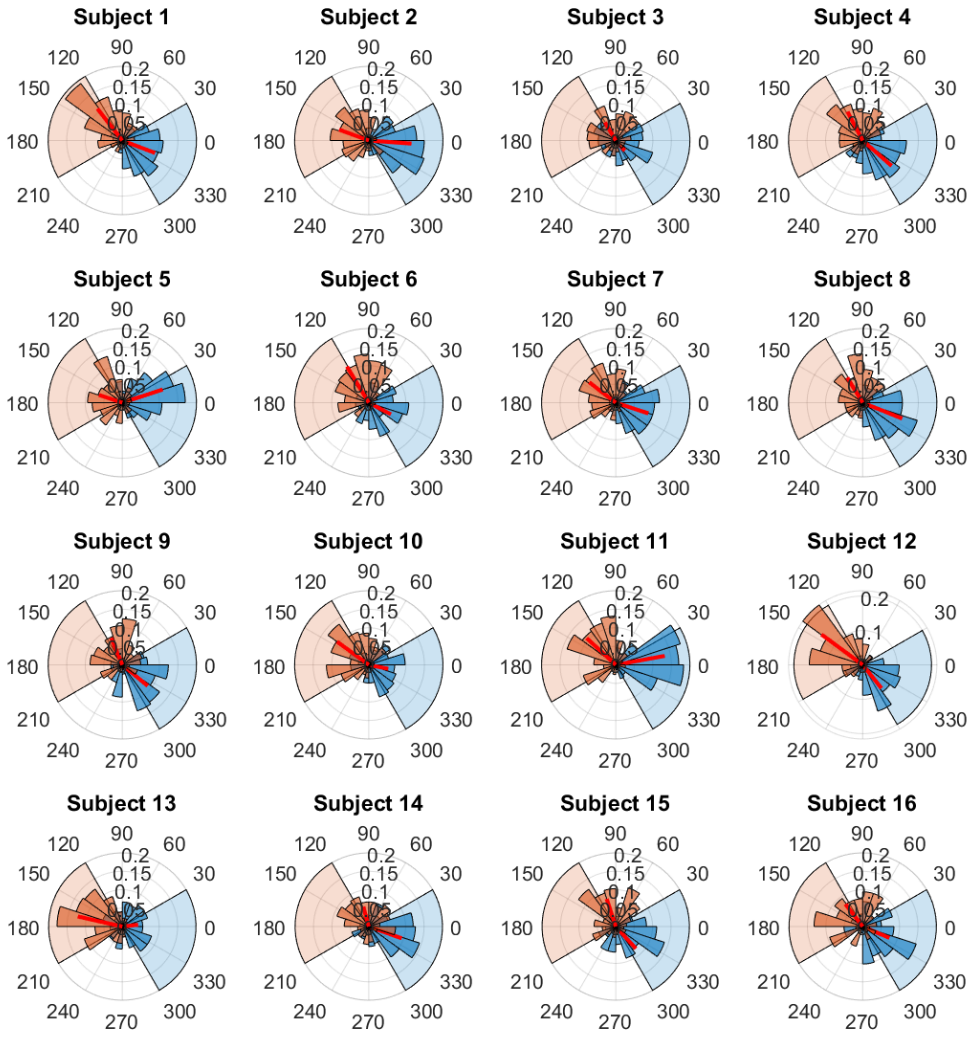

Phase accuracy for each subject at trial level. Up-state cues are shown in blue. Down-state cues are shown in red. Trial level phase accuracy for each individual subject shows a clear distinction between up- and down-state cues for all subjects.

Supplementary Figure 5: Oscillatory analysis of up- versus down-state cues.

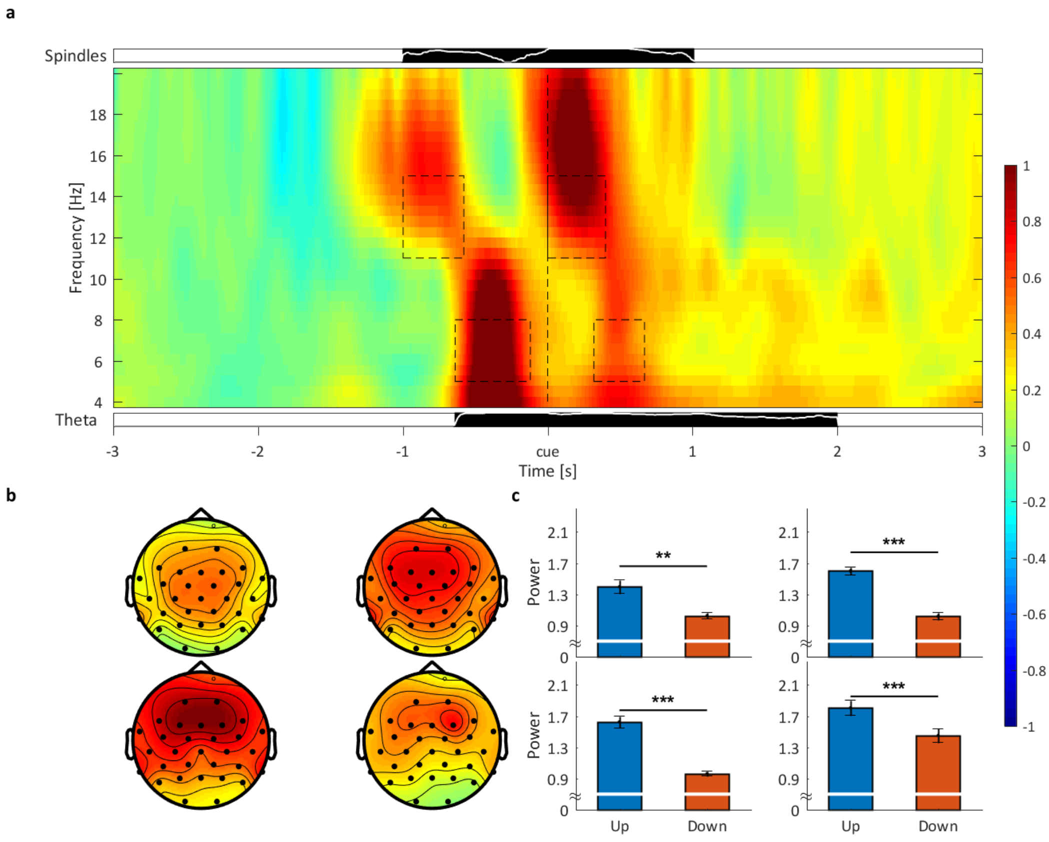

Oscillatory analysis of up- versus down-state cues. Panel a) illustrates the power contrast between words presented during SOs up- and down-states. Black bars (significant cluster in frequency band analysis) with white lines below and above the time-frequency plot indicate the number of significantly differing electrodes for the theta and spindle band respectively. The full height of the bar corresponds to 100% (31) electrodes. Panel b) topographical distribution of the areas marked with a dashed box in a) for the spindle (top row) and theta (bottom row) band, pre-stimulus (left column) and post-stimulus (right column). Significant electrodes are shown as filled black dots. Panel c) shows the same data averaged across time, frequency and significant channels within the respective cluster. The power is scaled between -1 and 1 for both panels a) and b). Time-frequency data is shown for the Fz electrode.

Supplementary Figure 6: Up-State Remembered versus Non-Remembered Words Time-Frequency Analysis

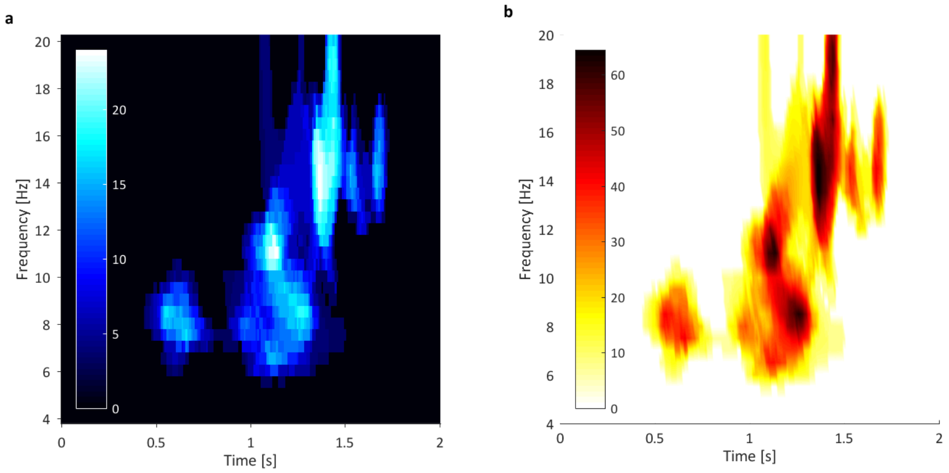

Positive cluster up-state remembered vs non-remembered. a) Number of electrodes involved in positive cluster found in time-frequency analysis of up-state remembered versus non-remembered words across frequency and time. b) Summed t-values for positive cluster found in time-frequency analysis of up-state remembered versus non-remembered words across frequency and time. The time-frequency analysis of up-state cues of remembered versus non-remembered words between 0 and 2 seconds after stimulus onset, across all channels and from 4 to 20 Hz revealed one significant positive cluster lasting from 0.44 to 1.73 seconds and involving all electrodes (P = 0.013).

Sleep stage specific EEG results

For memory cues played during the up-state in sleep stage N2 (n = 72.31 ± 11.72) we observed a significant increase in theta power for later remembered compared to later non-remembered words between 500 and 1730 ms involving a cluster of 29 channels (*P* = 0.022, see Supplementary Figure 7a and Figure 7b left column, bottom row). Also in the spindle band, the overall analysis revealed a significant increase in spindle power for remembered compared to non-remembered words between 920 and 1730 ms involving 30 electrodes (*P* = 0.021, see Supplementary Figure 7b left column, top row). In contrast to cues presented during SO up-states, we did not observe any significant power differences for remembered vs. non-remembered words played in the SO down-state, neither for the theta (no cluster found) nor the spindle band (no cluster found, see Supplementary Figure 7c and d). Even a more

restricted test-statistics limited to the time-range of the up-state clusters revealed no significant effect. In a next step we directly compared the oscillatory fingerprint of up- and down-state reactivation, by contrasting the 'subsequent reactivation effect' (i.e. remembered vs. non-remembered words) between those conditions (Supplementary Figure 7e and f). Here, an increase in spindle activity for up- as compared to down state cueing was observable, ranging from 920 to 2000 ms and involving all 31 electrodes (P = 0.005, see Supplementary Figures 7e and f top row). No significant difference emerged with regards to theta activity (P > 0.10, Supplementary Figures 7e and f bottom row).

During SWS, memory cues targeted into SO up-states showed only a trend for an increase in theta (P = 0.093, from 930 to 1480 ms) and no significant increase for spindles (P > 0.20, see Supplementary Figure 8a and b; overall number of cues: 231.38 ± 20.98). For cues presented during SO down-states there were no significant power differences for theta or spindles (both exhibited no significant clusters, see Supplementary Figure 8c and d). Contrasting the 'subsequent reactivation effect' also showed no significant difference (theta: P > 0.30, spindles: P > 0.60, see Supplementary Figure 8e and f).

When directly comparing memory cues played during the up-states of sleep stage N2 and SWS we observed a significant increase in theta power for later remembered words in N2 as compared to SWS across the whole range (0 - 2000 ms) involving all channels (P < 0.001, see Supplementary Figure 9a and 9b left column, bottom row). Also in the spindle band, the overall analysis revealed a significantly higher spindle power for words remembered during N2 compared to SWS between 300 and 2000

ms involving all electrodes (*P* = 0.003, see Supplementary Figure 9b left column, top row).

Supplementary Figure 7: N2 Oscillatory Results

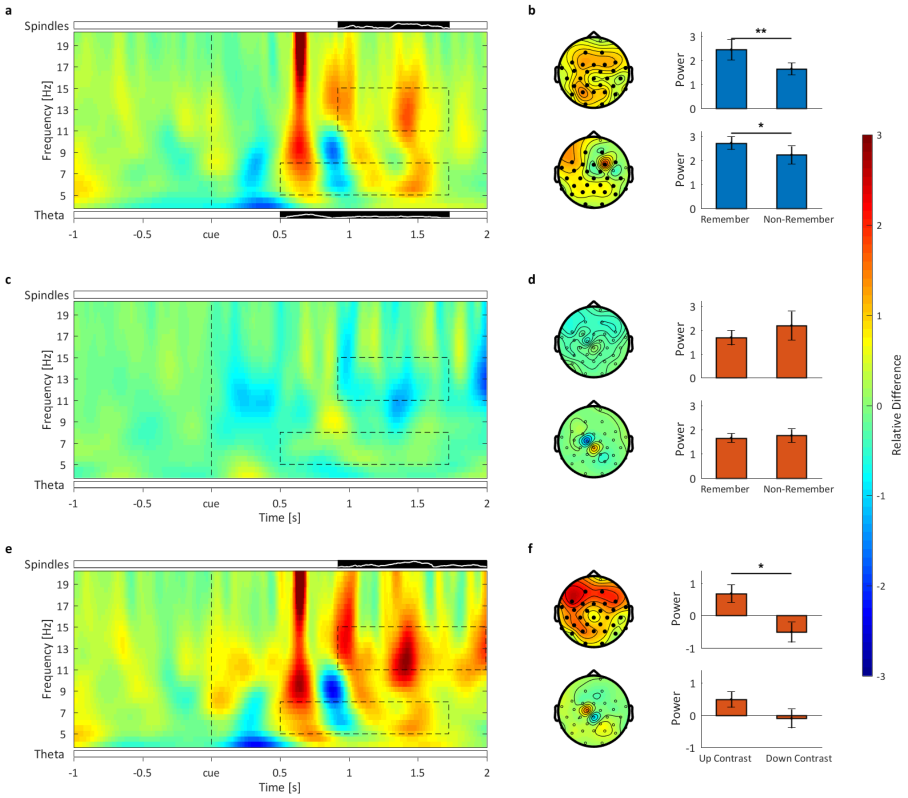

N2 Oscillatory Results. Time-frequency contrasts between remembered and not-remembered words in the theta and sleep spindle band for a) up-state and c) down-state cues, averaged over all 31 significant spindle electrodes. Black bars (significant cluster in frequency band analysis) with white lines below and above the time-frequency plot show the number of significantly differing electrodes for the theta and sleep spindle band respectively. The full height of the bar corresponds to 100% (31) electrodes. Dashed boxes indicate the areas of significant difference between remembered and not remembered words. These time-windows were used to illustrate the topographical distributions (b, d and f) left column, top row spindle band, bottom row theta band; significant electrodes shown as filled black dots). b), d) and f) right column show the mean power within the significant clusters, averaged over the significant electrodes, all frequencies and time in the sleep spindle (top) and theta (bottom) band. For up-state cueing a) remembered words show enhanced power in the theta (5-8 Hz) as well as the sleep spindle (11-15 Hz) range compared to not-remembered words. Averaged over time, channels and frequency band, within these clusters this difference was significant in the theta band ($t15 = 2.00$, $P = 0.032$; see b right column, bottom row) and in the spindle band ($t15 = 2.73$, $P = 0.008$; see b, right column, top row). For words presented during down-states c) no significant difference emerged between remembered and forgotten words, neither in the sleep spindle nor the theta band. Consequently, averaged activity in those clusters observed in the analysis of SO up-states did not reveal any significant differences for down-state cues, neither in the theta ($t15 = -0.35$, $P = 0.635$) nor the spindle band ($t15 = -1.50$, $P = 0.923$). The difference between the two

contrasts of up and down e) showed enhanced power in the spindle band, but not in the theta band. Averaged activity in the spindle cluster (t15 = 2.34, P = 0.017; see f right column, top row) showed a significant difference, while theta activity (averaged over the duration of the SO up-state theta cluster) showed no statistical difference (t15 = 1.44, P = 0.109; see f right column, bottom row). Mean ± s.e.m. are indicated. **: P < 0.01; *: P < 0.05.

Supplementary Figure 8: SWS Oscillatory Results

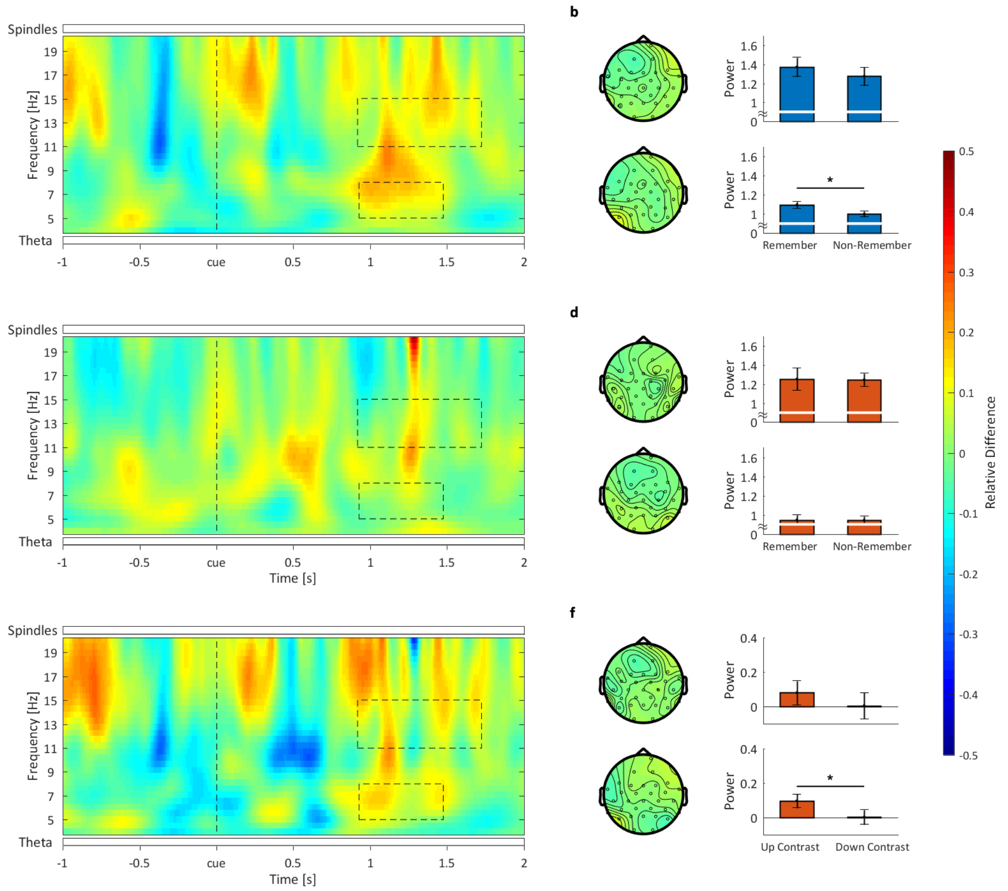

SWS Oscillatory Results. Time-frequency contrasts between remembered and not-remembered words in the theta and sleep spindle band for a) up-state and c) down-state cues, averaged over all 18 electrodes that show a trend in the theta band. As there were no significant clusters in SWS for spindle, we used the significant time windows and electrodes of the N2 clusters for b), d) and f) right column, showing the mean power, averaged over the electrodes, all frequencies and time in the sleep spindle (top) band. For the same analysis in the theta (bottom) band, we used the time window that showed a trend of a difference for up-state cueing. For up-state cueing a) remembered words show no enhanced power in the theta (5-8 Hz) nor the sleep spindle (11-15 Hz) range compared to not-remembered words. Averaged over time, channels and frequency band, within these clusters this difference was significant in the theta band (t15 = 2.43, P = 0.014; see b right column, bottom row) but not in the spindle band (t15 = 1.28, P = 0.109; see b, right column, top row). For words presented during down-states c) no significant difference emerged between remembered and forgotten words, neither in the sleep spindle nor the theta band. Consequently, averaged activity in those clusters observed in the analysis of SO up-states did not reveal any significant differences for down-state cues, neither in the theta (t15 = 0.05, P = 0.480) nor

the spindle band (t15 = 0.07, P =0.472). The difference between the two contrasts of up and down e) again showed no enhanced power in the spindle band, nor in the theta band. Averaged activity in the spindle cluster (t15 = 0.33, P = 0.374; see f right column, top row) showed no significant difference, but averaged theta activity showed statistical difference (t15 = 1.84, P = 0.043; see f right column, bottom row).

Supplementary Figure 9: Oscillatory Results N2 VS SWS

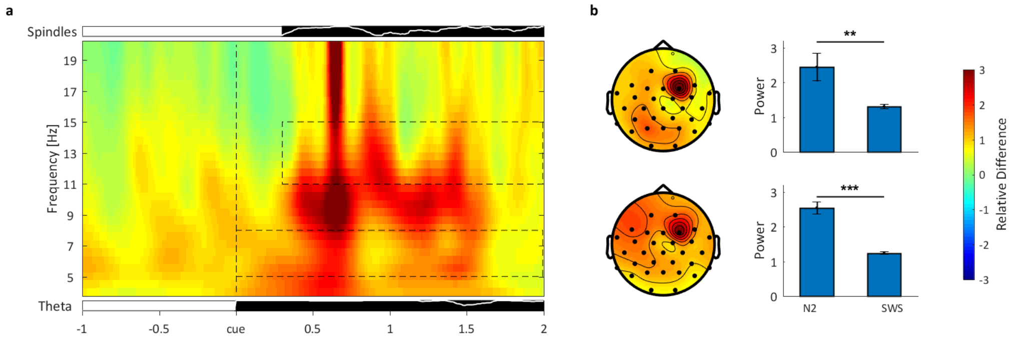

N2 vs. SWS Oscillatory Result. a) Time-frequency contrasts between up-state cued remembered words in N2 and SWS in the theta and sleep spindle band averaged over all electrodes. Dashed boxes show the duration of the significant clusters for theta (0 – 2000 ms) and spindles (300 – 2000 ms). These time windows were used to show the topographical distribution (b) left column) of theta (bottom) and spindles (top). b) right column shows the averaged relative difference between N2 and SWS averaged over time frequency and channels. We find a significant difference between N2 and SWS for both theta (t15 = 7.04, P < 0.001; b) bottom right) and spindles (t15 = 2.76, P = 0.007; b) top right). Mean ± s.e.m. are indicated. **: P < 0.01; ***: P < 0.001.

Supplementary Figure 10: ERP for 0.5 – 1 Hz Bandpass filtered Signal

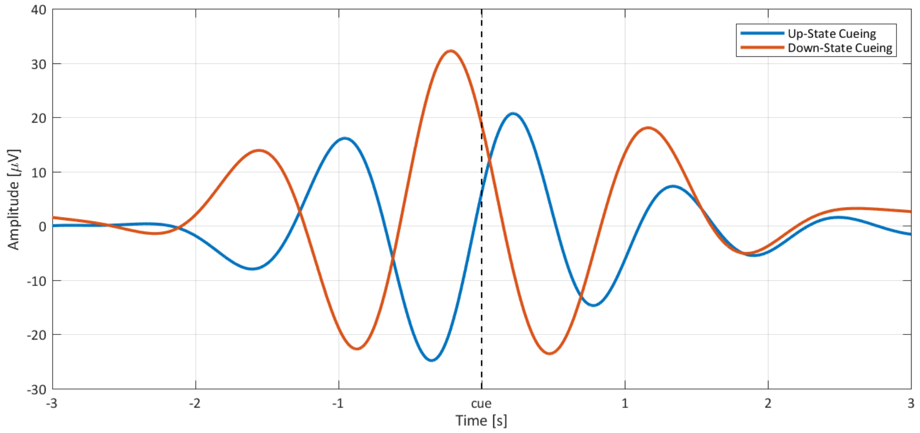

0.5 – 1 Hz bandpass filtered ERP. To validate whether the targeting algorithm (lowpass filtered at 1.5 Hz) captures the correct states of slow wave (0.5 – 1.0 Hz) the data was bandpass filtered between 0.5 – 1 Hz. Comparable to the main results (Figure 1*)* the ERP-analyses revealed that up-state cues were located at the down-to-up transition of the cortical slow wave (beginning of slow oscillatory up-state), and that down-state cues were played at the up-to-down transition (beginning of slow wave down-state), confirming that the slow wave detection algorithm does indeed detect the proposedly critical states of the slow wave. Data is shown for electrode Fz.
